# Organoids as a tool to study homeostatic and pathological immune–epithelial interactions in the gut

**DOI:** 10.1093/cei/uxad118

**Published:** 2024-03-29

**Authors:** Emma Højmose Kromann, Ainize Peña Cearra, Joana F Neves

**Affiliations:** Centre for Host Microbiome Interactions, King’s College London, London, United Kingdom; Centre for Host Microbiome Interactions, King’s College London, London, United Kingdom; Department of Immunology, Microbiology and Parasitology, Faculty of Medicine and Nursing, University of the Basque Country (UPV/EHU), Bilbao, Spain; Centre for Host Microbiome Interactions, King’s College London, London, United Kingdom

**Keywords:** immune cells, organoids, intestine, inflammation, infection, colorectal cancer

## Abstract

The intestine hosts the largest immune cell compartment in the body as a result of its continuous exposure to exogenous antigens. The intestinal barrier is formed by a single layer of epithelial cells which separate immune cells from the gut lumen. Bidirectional interactions between the epithelium and the immune compartment are critical for maintaining intestinal homeostasis by limiting infection, preventing excessive immune activation, and promoting tissue repair processes. However, our understanding of epithelial–immune interactions incomplete as the complexity of *in vivo* models can hinder mechanistic studies, cell culture models lack the cellular heterogeneity of the intestine and when established from primary cell can be difficult to maintain. In the last decade, organoids have emerged as a reliable model of the intestine, recapitulating key cellular and architectural features of native tissues. Herein, we provide an overview of how intestinal organoids are being co-cultured with immune cells leading to substantial advances in our understanding of immune–epithelial interactions in the gut. This has enabled new discoveries of the immune contribution to epithelial maintenance and regeneration both in homeostasis and in disease such as chronic inflammation, infection and cancer. Organoids can additionally be used to generate immune cells with a tissue-specific phenotype and to investigate the impact of disease associated risk genes on the intestinal immune environment. Accordingly, this review demonstrates the multitude of applications for intestinal organoids in immunological research and their potential for translational approaches.

## Teaser

This review discusses the significance of intestinal organoids as a model for studying immune-epithelial interactions in the gut. Organoids replicate key features of native tissues and have advanced our understanding of the immune system’s role in maintaining and regenerating the intestinal barrier during health and disease. The review highlights the various applications of intestinal organoids in immunological research, including co-culturing with immune cells, generating tissue-specific immune cells, and exploring the impact of disease-associated risk genes on the intestinal immune environment.

## Introduction

The intestinal mucosa is a dynamic site of interaction between host cells and environmental factors such as dietary metabolites, commensal microorganisms, and pathogens present in the gut lumen. A single layer of epithelial cells makes up the intestinal barrier, below which lies the lamina propria, a richly innervated and vascularised layer of loose connective tissue densely populated with immune cells, fibroblasts, and other stromal cells. Barrier integrity is essential for intestinal homeostasis and is safeguarded by constant, bidirectional communication between the epithelium and resident immune cells allowing for a rapid response to tissue damage and infection while maintaining tolerance to innocuous immune triggers. Robust *in vitro* cell culture systems are a necessary tool for untangling this complex network of interactions and complement the use of animal models. Early 2D models culturing intestinal epithelial cells on mesenchymal feeder cell layers are limited in their cellular complexity, cannot be maintained for prolonged periods, and have low spatial resolution which limits their physiological relevance and translatability to the *in vivo* gut environment [1]. The development of intestinal ‘organoids’, 3D stem cell-derived tissue cultures closely resembling the intestinal barrier, has therefore rapidly been picked up by immunologists to model immune interactions within the intestinal epithelium. Here, we review how co-culturing these organoids with immune cells has advanced our understanding of the immune–epithelial interplay in the gut during homeostasis, inflammation, infection, and cancer.

## Intestinal organoids as a model of the human intestine

**Figure 1.**
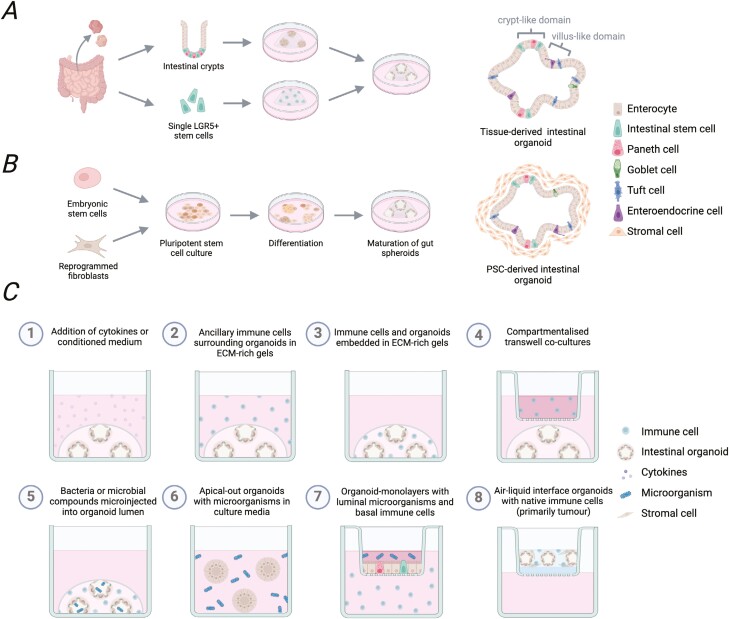
Generation of tissue- or PSC-derived intestinal organoids and reported immune-organoid co-culture approaches. Intestinal organoids are primarily generated following two approaches. (**A**) Tissue-derived intestinal organoids can be grown from isolated crypts or single Lgr5 + ISC to produce epithelial-only cell structures. (**B**) Embryonic stem cells or induced pluripotent stem cells can be pushed sequentially towards endoderm and gut differentiation to produce PSC-derived intestinal organoids with both epithelial and mesenchymal cell compartments. (**C**) Organoids can be used for immunological studies in several different ways. This includes addition of cytokines or immune cell conditioned media (1), co-culture with immune cells (2–4), inclusion of microbial components, (5–7) or as air-liquid interface organoids which co-develop native immune cells (8).Figure created with BioRender.com.

The intestinal epithelium primarily consists of absorptive enterocytes interspersed with specialized subsets of secretory cells including Paneth cells, tuft cells, goblet cells, and enteroendocrine cells. Lgr5^+^ intestinal stem cells (ISCs) reside in invaginated crypts along the entire intestine giving rise to all epithelial subtypes and continually replenishing the epithelial barrier [2]. In a 2009 landmark study, it was discovered that isolated small intestinal crypts or single Lgr5^+^ ISCs could be embedded in extracellular matrix (ECM)-rich gels and cultured long-term in growth factor-defined medium [3]. These culture conditions result in the formation of 3D cell clusters, which closely model the cellular heterogeneity of the intestinal epithelium and the architecture of the native intestine (Fig. 1A). Here differentiating epithelial cells grow out of basal stem cell crypts into the organoid center and retain apico-basal polarity creating an enclosed lumen inside the cell clusters. It has subsequently been demonstrated that organoids generated from distinct regions of the small intestine exhibit morphology and gene expression typical of their tissue of origin. Accordingly, duodenal, jejunal, and ileal organoids can each be cultured enabling the study of specific intestinal compartments [4, 5]. Although intestinal organoid cultures were first established using mice, protocols have since been adapted to generate small intestinal ‘enteroids’ or large intestinal ‘colonoids’ from human biopsy samples [6].

Building on a growing understanding of early development, intestinal organoids have also been generated from human pluripotent stem cells (PSC) by sequentially inducing endoderm formation before pushing cells toward gut patterning [7] (Fig. 1B). Resulting cell structures are then encapsulated in supportive gel matrices and maturated to achieve a small intestinal or colonic phenotype depending on the media composition [7, 8]. While these offer an advantage over tissue-derived organoids as they develop mesenchymal cells, which secrete native ECM around the epithelial structures adding a further layer of complexity, transcriptional analyses reveal PSC-derived organoids to more closely resemble a foetal rather than adult gut [9]. However, methods to promote their maturation have been described, including the incorporation of mechanical cues [10] or ISC niche factors [9, 11, 12]. Multilineage organoids can also be derived from intestinal tissues by culturing tissue fragments in collagenmatrices exposed to air [13]. While they represent an early organoid system with both epithelial and mesenchymal compartments, air–liquid interface organoids have not gained the same popularity and are primarily used in cancer research [14].

Protocols to increase specialised epithelial cell types including Paneth cells [15], goblet cells [15], and enteroendocrine cells [16] have been established and facilitate studies on the immune interplay with particular epithelial subsets. Moreover, microfold cells, capable of transferring luminal antigens across the epithelial barrier to underlying immune cells, derive from Lgr5^+^ ISC but are only detectable in organoids treated with the cytokine RANKL [17]. As a result, advances within the last decade have progressed gut organoid technology from proof-of-principle to a robust, customisable tool with great promise for fundamental and translational research.

## Applications for intestinal organoids in immunological research

Intestinal organoids provide a reductionist platform for interrogating immune interactions in the gut (Fig. 1C and Table 1). Addition of recombinant cytokines is a simple and extensively used approach to determining the impact of specific immune mediators on the intestinal epithelium [18]. The first report of a co-culture system for immune cells and intestinal organoids showed that primary T cells survive long-term culture with murine enteroids [19]. Several protocols have since been established for setting-up co-cultures with innate immune cells including neutrophils [20, 21], macrophages [21], and innate lymphoid cells (ILC) [22–24]. These immune cells can be embedded next to the organoids within the ECM-rich gels to allow contact-dependent interactions. Alternatively, immune and epithelial compartments can be separated using transwell inserts or supernatants from immune cell cultures can be spiked into the organoid media to determine only the effect of soluble mediators released from these cells.

**Table 1.** Summary of immune-organoid co-cultures

Type of immune cell or bacteria cell	Organoid type	Readout	Reference
*Study of immune impact on epithelial regeneration*			
Splenic CD4 + T cells	Mouse enteroids	ISC renewal and differentiation	Biton et al. (2018)
Jurkat T cells	Human PSC-derived small intestine	Organoid growth and epithelial maturation	Jung et al. (2018)
Lamina propria CD4^+^ T cells	Human enteroids	Organoid outgrowth	Schreurs et al. (2019)
Lamina propria ILC1	Mouse enteroids Human PSC-derived small intestine	Organoid crypt budding, ECM remodelling	Jowett et al. (2021)
Mesenteric lymph node ILC2	Mouse enteroids	Goblet cell differentiation	Waddell et al. (2019)
Splenic CD4 + T cells	Mouse enteroids	Organoid viability	Joly et al. (2015)
Splenic CD4 + and CD8 + T cells	Mouse enteroids	Organoid damage and epithelial differentiation	Eriguchi et al. (2018
Peripheral CD4 + and CD8 + T cells	Mouse enteroids Human enteroids	Organoid outgrowth	Takashima et al. (2019)
Bone marrow-derived macrophages	Mouse enteroids	Epithelial resistance and permeability	Spalinger et al. (2020)
Peripheral T cells	Mouse enteroids Human enteroids	Organoid viability	Matsuzawa-Ishimoto et al. (2020)
Small intestine intraepithelial lymphocytes Peripheral T cells	Mouse enteroids Human enteroids	Organoid crypt budding, viability and Paneth cell numbers	Matsuzawa-Ishimoto et al. (2022)
Splenic and colonic CD11c + leukocytes, bone-marrow-derived dendritic cells	Mouse enteroids	Organoid morphology and epithelial differentiation	Ihara et al. (2018)
Lamina propria lymphocytes and ILC3	Mouse enteroid	Organoid crypt buds and proliferation	Lindemans et al. (2015)
BMM-derived dendritic cells	Mouse enteroid	Viability	Jones et al. (2019)
*Study of epithelial impact on immune cell behaviour*			
Peripheral T cells	Mouse enteroid	T cell proliferation and morphology	Rogoz et al. (2015)
Splenic CD4 + T cells	Mouse enteroid	T cell proliferation	Biton et al. (2018)
Bone marrow or peripheral ILC precursors	Mouse enteroid Human PSC-derived small intestine	ILC expansion, expression of transcription factors, surface markers and cytokines	Jowett et al. (2022)
PSC-derived monocytes	Human PSC-derived small intestine	Macrophage morphology and cytokine secretion	Tsuruta et al. (2022)
Monocyte-derived macrophages	Human enteroid monolayer	Macrophage morphology	Noel et al. (2017)
Peripheral polymorphonuclear neutrophils	Human enteroid monolayer	Surface marker expression and cytokine production	Lemme-dumit et al. (2022)
*Study of host–microbial interactions*			
CD11c^+^ DCs and UC-irradiated *Lactobacillus* reuteri or *L. reuteri*-conditioned media	Mouse Colonoids	IL-10 production	Engevik et al. (2021)
Macrophages and *Lactobacillus murinus*	Mouse enteroid monolayer	LDH levels and expression of genes encoding tight junction proteins	Hu et al. (2022)
Human monocyte-derived macrophages and *Escherichia coli*	Human enteroid monolayer	Monolayer integrity and cytokine production	Noel et al. (2017)
Polymorphonuclear neutrophils and *Shigella*	Human enteroid monolayer	Development of a PMN co-culture model	Lemme-Dumit et al. (2022)
Intraepithelial Ly6A + CCR9 + CD4 + T cells	Mouse enteroid	Development of T-cell co-culture system	Parsa et al. (2022)
*Study of colorectal cancer immunology*			
Tumour-reactive T cells	CRC patient-derived tumour colonoids	Development of patient-derived tumour organoid-T-cell coculture system	Dijkstra et al. (2018)
Tumour infiltrating lymphocytes	Mouse and CRC patient-derived tumour colonoids	Development of an air-liquid interface (ALI) organoid model	Neal et al. (2018)
T cells	Mouse colonoids	Development of engineered murine CRC organoids to assess neoantigen expression	Westcott et al. (2022)
Intraepithelial lymphocytes	Mouse APC KO enteroid	Antitumour immune response	Morikawa et al. (2021)
IL-2-activated NK cells-conditioned medium	Human CRC patient-derived tumour colonoids	Apoptosis of tumour cells	Parseh et al. (2022)
BMM-conditioned medium	Mouse enteroid	Self-renewal and proliferation of ISCs	Saha et al. (2016)

Epithelial–immune interactions in the gut are affected by the presence of dietary factors and microorganisms, which can be included in organoid models to add an additional layer of complexity. Microinjection of metabolites or live bacteria into the organoid lumen offers huge potential for the study of these interactions in a physiologically relevant localization [25]; however, this is time consuming, can result in heterogenous exposure, and has limited scalability. Instead, organoid monolayers can be grown in transwell inserts to allow for the compartmentalisation of basal immune cells and environmental factors on the luminal side [20, 26]. ‘Inside-out’ intestinal organoids also exist as an alternative, making the luminal surface more accessible while maintaining the dimensionality of whole organoids [27]. Organoids with reversed polarity are easily generated by transferring tissue-derived organoids into suspension, which causes the apico-basal polarity of the epithelial layer to switch while retaining integrity of the epithelial barrier [27]. This allows researchers to determine the direct impact of luminal factors on the epithelium by simply adding them into the culture media, retaining homogenous exposure across organoids. Therefore, apical-out organoids provide an alternative *in vitro* model that enables the study of nutrient uptake, drug absorption and metabolism and can be useful to study epithelial barrier function and host-pathogen interactions, which are relevant in gastrointestinal-related diseases.

## Generating tissue-specific immune cells using intestinal organoids

A key advantage of co-cultures between intestinal organoids and immune cells is that they allow us to gain insight into the tissue specification of immune cells residing in hard-to-access tissues. The first report of co-cultures between intestinal organoids and immune cells found that murine peripheral blood T cells acquired membrane projections, expression of gut homing markers, and integrated within the epithelium [19]. Similarly, the development of tissue-specific phenotypic characteristics has also been observed across multiple innate immune cell types including neutrophils, macrophages, and ILCs [20, 24, 28, 29].

ILCs make up a tissue-resident immune cell population present in mucosal tissues, which have gained recognition for their role in protective immunity and barrier integrity in homeostasis and infection [30]. A circulating ILC precursor can be isolated from blood and used to generate mature ILCs *in vitro* [31]. Previously, this relied on modified bone-marrow derived stromal feeder layers and required specific stimulatory cytokines to steer precursor differentiation towards a specific ILC subset [31, 32]. Using PSC-derived small intestinal organoids to limit variability from donor age, genetics or disease status, ILC precursors expand manifold and differentiates into ILC that recapitulate the transcription factor, surface marker and cytokine profile of mature human intestinal ILCs without the need for additional stimulatory cytokines [24]. Remarkably, only the organoid epithelial compartment and not the stromal compartment was required to maximize expansion and achieve gut-like ILC subset distribution, indicating that *in vivo* ILC maturation relies more on the intestinal epithelium than previously thought [24].

These studies start to uncover the potential of intestinal organoids to generate tissue-resident immune cells. Using organoid-based platforms for immune cell differentiation mitigates reliance on invasive procedures to gain access to primary immune cells and can generate high numbers of rare cell populations for experimental purposes and for autologous immune cell therapies, as it has been explored with thymic organoids [33].

## Immune cells in the intestinal stem cell niche

ISCs are found at the base of intestinal crypts, surrounded by a multicellular niche providing local signaling factors to promote stem cell maintenance such as Wnt and Notch ligands [34]. Within the murine small intestine, CD4^+^ helper T cells can be found adjacent to Lgr5^+^ ISC within small intestinal crypts [35]. Here, bidirectional communication between the cell types has important consequences for the regulation of ISC renewal and differentiation. Organoid cultures have played an essential role in advancing our understanding of how this occurs. Early co-cultures revealed that T cells cluster around enteroids [19], allowing this system to be exploited by Biton et al. in order to examine the impact of interactions between ISCs and T cells [35]. By co-culturing differently polarized lamina propria CD4^+^ T cells, or their canonical cytokines, with murine small intestinal organoids, they observed that pro-inflammatory helper type 1 T cells (T_H_1), T_H_2, and T_H_17 were found to reduce the ISC population while anti-inflammatory induced regulatory T cells promoted stem cell maintenance [35]. Expression of major histocompatibility II complexes (MHC-II) by ISC is well established in both human and mouse but limited to the small intestinal epithelium during steady state [36]. However, studies indicate that interferon-γ (IFNγ), increased in inflamed intestines, can increase MHC-II expression and antigen processing capacity in human enteroids [37]. A role for T-cell-mediated epithelial maturation has also been established as a result of organoid models. Transcriptomic analysis of PSC-derived intestinal organoids revealed the epithelial compartment of these to be less mature than tissue-derived intestinal organoids [11], instead, more closely resembling that of fetal tissues [9]. However, co-culture with Jurkat T cells promoted their transcriptional and functional maturation in an IL-2-dependent manner, thus increasing their similarity to the adult intestine [11]. Moreover, TNF-α derived from effector memory CD4^+^ T cells was shown to regulate epithelial development of human fetal tissue-derived intestinal organoids in a dose-dependent manner [38].

Together, these studies showcase the capacity of a reductionist *in vitro* system to untangle the role of different immune cells in maintaining the ISC population.

## Commensal and pathogenic microorganisms impact on intestinal immune homeostasis

The bidirectional interactions between the gut microbiota and the host are essential for maintaining intestinal homeostasis [39]. However, microbial changes such as decreased microbial diversity in chronic inflammation or as a result of broad-spectrum antibiotics can render the host more susceptible to pathogen colonization [40]. Given that questions about how commensal and pathogenic microorganisms influence the host immune system and barrier function remain unanswered, organoids have been used in diverse forms (Fig. 1C) to address those questions [41].

**Figure 2.**
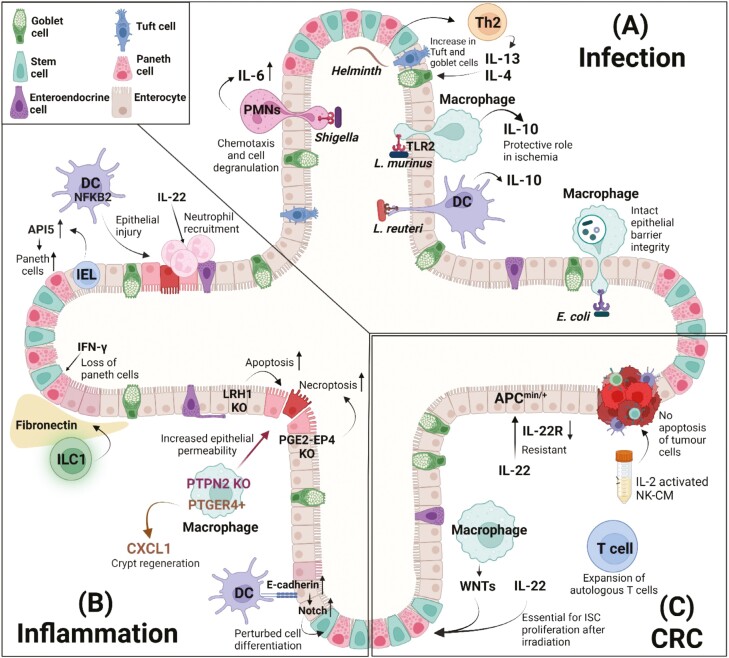
Overview of the data obtained from mouse and human co-cultures of intestinal organoids with immune cells, bacterial cells or cytokines in models of infection, inflammation and colorectal cancer (CRC). (**A**) For infection studies, organoid monolayers grown in transwell cell culture inserts are usually used. Co-culture of organoids with commensal strains such as *Lactobacillus murinus* and *Lactobacillus reuteri* showed the production of the anti-inflammatory IL-10 by DCs and macrophages in the intestine. The role of Helminths and other bacteria such as *Shigella* and *E. coli* has also been studied in an organoid-coculture model. (**B**) To study the contribution of a specific gene to intestinal inflammation development, many studies have used genetically engineered mice to generate intestinal organoids (epithelial cells deficient in LRH1 and PGE2-EP4) or to obtain genetically modified immune cells including dendritic cells (NF-kB2 KO) and macrophages (PTPN2 or PTGER4 + KO macrophages). Organoids have also been challenged with cytokines such as IL-22 and IFN-γ and co-cultured with immune cells including ILC1s, dendritic cells, macrophages, and intraepithelial lymphocytes (IELs) to analyse how specific cells or cytokines impact the epithelial barrier function thereby promoting intestinal inflammation. (**C**) For CRC studies, organoids established from Apc^Min/+^ murine model and patient-derived tumour organoids are normally applied. To increase the understanding in which molecules or cells drive or prevent tumorogenesis co-culture systems of organoids with immune cells such as IELs and macrophages, cytokines (IL-22) and conditioned medium derived from NK cells have been described. Furthermore, a modified T-cell co-culture system with patient-derived tumour organoids has been developed to expand and select patient-specific tumour reactive cells T-cells *ex vivo*. Figure created with BioRender.com.

*Lactobacillus* is a commensal genus of bacteria which promote multiple beneficial processes in the gut, including in the maintenance of intestinal homeostasis [42]. One potential mechanism for their protective action is through their promotion of an anti-inflammatory environment. A recent study microinjected UV-irradiated *Lactobacillus reuteri*, or conditioned media, into mouse colonoids revealing that their presence promoted the production of the anti-inflammatory cytokine IL-10 by dendritic cells mediated via toll-like receptor 2 [43] (Fig. 2A). Furthermore, co-culture of *Lactobacillus murinus* treated macrophages with organoids attenuated hypoxia-reoxygenation injury thus eliciting a protective effect on the intestinal epithelium [44] (Fig. 2A).

Immune responses to infection can also direct the specification of the intestinal epithelium. Helminth infection induces a strong type 2 immune response driving an increase in secretory tuft cells and goblet cells to promote worm expulsion [45–47] (Fig. 2A). Studies using murine enteroid models revealed that these effects are mediated directly on the epithelium by the pro-inflammatory cytokines IL-4 and IL-13 [45–48] (Fig. 2A) and excluded an autocrine role for IL-33 released by damaged epithelial cells or stromal cells in promoting the differentiation of secretory epithelial cells [46, 48]. Instead, IL-33 was found to promote IL-13 production by a heterogenous mesenteric lymph node immune population, hereby promoting goblet cell maturation indirectly [48].

Several viruses and bacteria can infect the intestinal tract causing gastroenteritis. These include enteric adenoviruses, which cause rapid accumulation of T cells in the intestinal epithelium upon infection [49]. Co-culture of murine enteroids infected with fluorescence-tagged adenovirus demonstrated that intraepithelial CD4^+^ T cells directly interact with infected cells promoting viral clearance [49]. Epithelial cells express the adenovirus receptor on their basolateral side, which is exposed on the outside of the 3D structures, and organoids can therefore be infected by a simple addition of virus to the culture media. In other cases, the enclosed organoid lumen can be made more accessible to pathogens by culturing 2D enteroid-derived monolayers on transwell inserts allowing for co-culture with apically accessible microbes and basally situated immune cells [50] (Fig. 1C). This 2D system was used to assess the influence of two distinct pathogenic *E. coli* strains on immune–epithelial interactions. Exposing the apical side of the intestinal organoid to the bacterial strains was found to cause macrophages to initiate an inflammatory response and extend their dendrites through the epithelial organoid layer, promoting pathogen clearance without compromising epithelial integrity [50] (Fig. 2A). Later, this system was harnessed to assess the impact of pathogenic *Shigella* bacteria on polymorphonuclear neutrophils [20]. The addition of *Shigella* increased expression of chemotaxis markers in neutrophils, resulting in the transmigration of PMNs across the epithelial barrier to eliminate the bacteria (Fig. 2A).

## Immune-mediated damage of the intestine

While immune cells are critical for the regulation of epithelial maintenance and the prevention of infection, prolonged inflammation, or aberrant immune reactions can also damage intestinal tissues as evidenced by graft-versus-host disease (GVHD) [51, 52], infection [53], and inflammatory bowel disease (IBD) [54]. Organoids have played an instrumental role in detangling the interactions between immune cells and the epithelium that drive these pathologies.

Haematopoietic stem cell transfer can be a life-saving procedure in patients with malignancies such as myeloid leukaemia. However, donor T cells often recognize host tissues as foreign resulting in GVHD. Much of the morbidity and mortality associated with GVHD can be attributed damage to the gastrointestinal tract [55]. Co-cultures of tissue-derived organoids with T cells from a non-identical allogeneic donors can be used to effectively model GVHD *in vitro* [51, 52, 56]. Crypts isolated from mice receiving allogeneic T-cell transfers exhibit impaired organoid-forming capacity indicating a loss of ISC fitness [51]. In line with this, allogeneic T cells co-cultured with murine enteroids and colonoids reduced LGR5^+^ gene expression and organoid viability [51, 57]. However, immunorecognition of the organoid epithelium was not found to be a requirement for T-cell-mediated damage. Once T-cell activation has occurred both allogeneic and syngeneic T cells impair organoid formation and survival [51, 57].

Activated immune cells have been shown to primarily damage intestinal organoids via secretion of pro-inflammatory cytokines such as TNF-α and IFN-γ [4, 51, 56, 57]. This was found to be partially mediated by NF-κB2 signalling using NF-κB2 deficient murine enteroids which exhibited resistance to TNF and IFN-γ-induced epithelial apoptosis [4]. Similarly, bone-marrow-derived dendritic cells (BMDCs) activated by bacterial lipopolysaccharide decreased viability of murine enteroids in an NK-κB2-dependent way [4] (Fig. 2B). In addition to causing a loss of Lgr5^+^ ISC [51, 57], IFN-γ impacts secretory Paneth cells which also reside in intestinal crypts [57, 58]. Important regulators of crypt stability, Paneth cells confer immunity to infection by secreting antimicrobial compounds [58], help select the microbial composition of the gut [59], and provide signalling cues for ISC maintenance [34]. IFN-γ treatment of murine enteroids caused rapid release of granules containing antimicrobial peptides, quickly followed by Paneth cell apoptosis and extrusion into the organoid lumen [57, 58]. This resulted in a loss of the majority of functional Paneth cells upon 24 hours of IFN-γ treatment [58]. IFN-γ also can also increase permeability of murine enteroids by stimulating cleavage of epithelial cell–cell junctions [60]. Thus, organoids have demonstrated widespread effects of pro-inflammatory signals on barrier maintenance in the gut.

Chronic inflammation of the intestines is a characteristic feature of IBD, which primarily comprises Crohn’s disease (CD) and ulcerative colitis (UC). While the etiology of IBD is undetermined, dysfunctional intestinal immune–epithelial interactions play a central role in disease development and progression. Multiple immune cell types orchestrate intestinal inflammation in patients with this disease. In addition to T cells, ILC1s, which are found in low numbers in homeostatic intestinal tissues, accumulate in inflamed intestines of CD patients [61]. A study using PSC-derived organoids found that human ILC1s secrete TGF-β to regulate both epithelial and mesenchymal cell behaviour [23]. This was only observed for ILC1s isolated from inflamed intestines of CD patients, which hyperproliferated in culture, and not from those isolated from uninflamed controls. As the organoids employed for this research were genetically, epigenetically, and environmentally identical, this demonstrated the ability of chronic inflammation to produce long-term alterations in immune cell phenotypes even after their removal from the inflammatory environment [23]. Remodelling of the ECM underlies many IBD-related sequalae. ILC1 increased ECM deposition by organoid associated fibroblasts thus demonstrating a functional link between increased ILC1 frequencies and IBD pathology [23] (Fig. 2B). Furthermore, aberrant interactions with the intestinal epithelium were also described upon TGF-β depletion on CD11c^+^ lamina propria leukocytes or BMDCs [62]. These adhesive interactions were mediated by E-cadherin expressed on BMDCs followed by Notch signalling activation in the intestinal epithelium (Fig. 2B), which led to the formation of undifferentiated cystic enteroids that lacked goblet cells [54]. In line with the results obtained in organoids, phagocytes expressing E-cadherin exhibit a pro-inflammatory phenotype, accumulate in the inflamed tissues of UC patients and mediate immune attachment to epithelial cells [54]. Like immune cells, epithelial cells express pattern recognition receptors, which detect pathogen- or damage-associated molecular patterns resulting in the activation of signalling cascades, including that of interferon regulatory factors (IRFs). Previously IRFs were thought to drive the upregulation of a protein known as interferon-stimulated gene 15 (ISG15) primarily in immune cells; however, recent work using human colonoids found these were additionally ably to produce ISG15 following exposure to the immunostimulant poly(I:C) and TNF-α [63]. This may have important implications for the role of the intestinal epithelium in the pathogenesis of IBD as leukocytes stimulated with ISG15 produced IBD-related cytokines [63].

Inflamed tissues from patients with IBD also have increased expression of the cytokine IL-22 [64] and genes induced downstream of IL-22 signalling [65]. However, the role of IL-22 in barrier homeostasis remains controversial. Early reports demonstrate that co-culture of IL-22-producing ILC3 with intestinal organoids and treating single LGR5^+^ ISC with IL-22 promotes organoid formation and growth [66]. This was shown to be protective against radiation injury as IL-22 pre-treated single LGR5 + ISC exhibited improved viability and organoid forming potential after *ex vivo* radiation compared with untreated crypts [66] (Fig. 2C). IL-22 also increases gene expression of epithelial junction proteins [67] and antimicrobial factors in existing Paneth cells [65, 66], further supporting the capacity of this cytokine to promote intestinal regeneration and barrier function. It was later determined by several independent groups that the IL-22-mediated increase in enteroid size is paralleled by a time- and dose-dependent decrease in organoid forming efficiency [67–69]. This is attributed to the growth of a highly proliferative transit amplifying cell compartment at the expense of maintaining ISC renewal [68, 69]. It cannot be precluded that IL-22 impacts small and large intestinal regions differently as the initial study showing a positive role for IL-22 in homeostasis used a combination of small and large intestinal tissues to generate their organoids [66] while subsequent experiments were performed solely on small intestinal enteroids [67–69]. Apart from the protective role that IL-22 has in the gut, gene expression analysis of colonoids treated with IL-22 shows an increase in endoplasmic reticulum (ER) stress, which is associated with increased susceptibility to inflammation [65]. This is supported by IL-22 ablation decreasing ER stress-related transcripts and improving histological disease scores in a microbiota-induced mouse model of inflammatory bowel disease [65]. Proving the manifold impacts of IL-22 on the intestinal epithelium, cytokine treatment of human colonic organoids increased expression of neutrophil chemotaxis molecules, a potentially harmful pathway in IBD [70, 71].

Overall, these studies show that the co-culture of intestinal organoids with immune cells or cytokines provide an excellent platform to improve our understanding of disease mechanisms and find potential therapeutic targets.

## Organoid-based study of genetic risk factors in intestinal inflammation

Intestinal organoids have been widely used to determine the impact of extrinsic factors regulating epithelial renewal in the gut. However, they also offer an attractive platform to investigate how endogenous gene expression contributes to intestinal homeostasis. Genome-wide association studies have indicated several genetic variants that confer susceptibility to intestinal inflammation and barrier dysfunction [72–74] but deciphering the functional consequences of these polymorphisms has proven challenging.

Chronic intestinal inflammation is considered multifactorial with both genetic and non-genetic factors contributing to disease onset and progression. More than 200 risk loci have been identified in patients with IBD and genetic predisposition to intestinal inflammation can in large part be attributed genetic variance in loci encoding autophagy related genes including *NOD2*, *IRGM*, and *ATG16L1* [72–74]. Autophagy is a widely conserved process that mediates the orderly degradation and recycling of cellular components. In addition to promoting IBD susceptibility, defects in the autophagy machinery renders epithelial cells more vulnerable to infection and immune-mediated damage [53, 56, 75, 76]. A fundamental requirement for epithelial autophagy is highlighted by small intestinal organoids generated from mice with *Atg16l1* selectively ablated in the intestinal epithelium. These organoids display decreased viability and Paneth cell numbers even in the absence of immune mediators [75]. Additionally, autophagy deficient organoids are more susceptible to immune-mediated damage as their culture with activated T cells resulted in smaller and less viable organoids compared to wild-type counterparts [56, 75, 77]. Enteroids generated from human donors homozygous for a common CD-associated *ATG16L1*^T300A^ variant are similarly vulnerable to pro-inflammatory stimuli [56], demonstrating a conserved reliance on epithelial autophagy during intestinal inflammation in both mice and humans. While pro-inflammatory stimuli can exacerbate the decrease in organoid fitness brought on by defective autophagy, unconventional γδ intraepithelial lymphocytes can restore viability and Paneth cell proportions in *Atg16l1* deficient enteroids by secreting the anti-apoptotic factor API5 [75] (Fig. 2B). The API5 effect was further confirmed with organoids derived from individuals homozygous for the IBD risk allele *ATG16L1*^T300A^ where API5 rescued organoid viability and Paneth cell numbers. While polymorphisms in autophagy-related genes have a well-established role in increasing IBD risk, organoid studies have also demonstrated impact of other risk genes on the epithelial-immune interplay. Ablation of *Lrh1* in murine enteroids, which encodes a nuclear receptor expressed throughout the intestinal epithelium, markedly reduced organoid viability and increased susceptibility to apoptosis when organoids were challenged with TNF [78].

Variance in immune cell intrinsic gene expression can also cause aberrant immune–epithelial interactions in the gut. Loss-of-function mutations in the *PTPN2* gene elevate the risk of developing several inflammatory disorders [74]. By co-culturing enteroids with monocyte-derived macrophages from patients with disease-associated PTPN2 polymorphisms (Spalinger et al. 2020) demonstrated that such loss-of-function mutations can result in an increased permeability of the intestinal epithelium thus diminishing barrier integrity [79] (Fig. 2B). Likewise, CXCL1 secreted by PTGER4 + macrophages was found to play a major role in epithelial regeneration in murine enteroids by increasing the number of proliferating cells and crypt budding efficiency [80]. In accordance with this research, loss-of-function mutations in *PTGER4* have also been linked to IBD, putatively due to impaired intestinal barrier resistance [81].

These studies demonstrate that intestinal organoids provide an excellent platform to validate susceptibility genes determined by genetic studies and facilitate investigations of the functional impact of disease associated genetic variation.

## Organoid models to study immune interactions in colorectal cancer

Colorectal cancer (CRC) originates from neoplastic epithelial cells in the gut and is one of the most common intestinal malignancies with increasing incidence due to environmental and genetic risk factors [82]. Immune evasion is a cancer hallmark and CRC organoid models can demonstrate how immune–epithelial interactions might be altered in bowel cancer. For example, CRC organoids can be generated from malignant patient tissue samples [14, 83] or by inactivating an allele of the tumor suppressor gene adenomatous polyposis coli (APC^min/+^) in mice, which is commonly found mutated in colorectal cancer patients [84].

Immune cells including NK cells play a major role in the tumour immunosurveillance; however, their effector function can be diminished in CRC. Therefore, understanding tumour immunosurveillance with organoid systems has come up as an invaluable tool to find novel treatment strategies. While epithelial susceptibility to pro-inflammatory signals has consistently been reported [4, 51, 56, 57], patient-derived CRC organoids demonstrated unaltered viability when exposed to conditioned media from natural killer cells rich in IFN-γ, TNF, and other pro-apoptotic molecules [83] (Fig. 2C). APC-ablated organoids also had decreased IL-22 receptor expression and were resistant to IL-22 treatment [85], demonstrating another change to the immune-epithelial interplay in CRC (Fig. 2C).

Tumours with low mutational burden express fewer tumour-specific antigens and evade immune surveillance more easily. CRC organoids with varying levels of tumour-specific antigens have been generated by transformation of murine colonoids with lentiviral viruses harbouring several CRC-typical mutations [86]. Low neoantigen organoids elicited a slower, weaker immune response when the mutated organoids were injected into the intestinal mucosa of live mice. This was due to insufficient cross-priming of cytotoxic CD8^+^ T cells that became exhausted and lost effector function.

Organoids can also inform treatment approaches to CRC. Radiation is a common treatment for most solid cancers but carries the risk of injuring surrounding tissues. Considering the critical role of an intact barrier in the maintenance of intestinal homeostasis, repair of damaged tissue is essential following radiation injury. Organoid studies have demonstrated that the presence of IL-22 in the intestine prior to injury plays a central role in epithelial regeneration following irradiation [66] alongside the secretion of Wnt by intestinal macrophages [87] (Fig. 2C).

Immunotherapy boosting host immune responses to malignant cells have proven itself a valuable addition to traditional therapies. Adoptive T-cell therapies, where patient-derived T cells are expanded in culture and transferred back into their original host, are an example of a currently employed cancer immunotherapy. Recent work has demonstrated how patient-derived tumour organoids are advancing treatments by selectively expanding autologous tumour-reactive T cells [88, 89] (Fig. 2C). Moreover, complex patient-derived organoids containing both native tumour infiltrating immune cells and stromal cells in addition to the neoplastic epithelium can be generated using an air–liquid interface approach [14]. While currently restricted by their limited lifespan, the inclusion of the surrounding tumour microenvironment in these organoids make them attractive prospects for precision cancer therapies. Taken together, this body of research on immune-CRC co-culture systems demonstrates their promise as an *ex vivo* platform for cancer research, promoting the translation of scientific discoveries to clinical practice.

## Conclusion and future perspectives

Intestinal organoids provide a versatile platform for modelling immune–epithelial interactions in the gut and have greatly advanced our understanding of the impact of immune cells and their mediators on the intestinal epithelium. Tissue-derived organoids from patients or transgenic mice are already being used to study disease-related epithelial defects and it was recently discovered that reprogrammed fibroblasts sampled from UC patients retain epigenetic imprinting leading to PSC-derived organoids with disease traits [90]. Furthermore, CRISPR-based technologies allow genome editing of induced PSC lines [91] and have recently been optimized to enable conditional knockdown or overexpression of target genes in tissue-derived organoids [92]. These discoveries reveal new possibilities for developing disease-specific human intestinal organoids and *in vitro* modelling immune–epithelial interactions in pathology. While less explored, organoids can also provide insight into tissue specification of immune cells in the gut. It will be of interest whether these disease models can offer insight into the impact of epithelial dysfunction on immune phenotype and potentially be used to generate disease-associated immune cells *in vitro.* Furthermore, patient-derived organoids have also emerged as a powerful model to study genetics, cellular, and molecular mechanisms behind CRC as they are easy to manipulate and closely mimic human CRC tumors [93]. However, there is a lack of detailed characterization in terms of the proportion and function of epithelial cells in CRC organoids, which can lead to the misinterpretation of the results. Furthermore, the low number of viable tumor stem cells present in the fresh biopsies hinders organoid generation [94]. Despite the limitations, the use of these organoids can potentially shed light on how patients will respond to the treatment thereby promoting the implementation of personalized medicine in the treatment of CRC [93].

The lack of specialized equipment required for organoid culture has facilitated the adoption and application of this technology within many areas of research. Despite their tremendous potential, it is important to keep in mind that findings from organoid co-culture studies are also heavily impacted by the intestinal region and developmental stage the organoids reflect. This might contribute to discrepancies between studies, and it is worth considering which type of intestinal organoids represents the most physiologically relevant model for a given research question. Variability in media composition between different research groups due to high cost of commercially available reagents or co-culture optimization might also cause unforeseen variation between studies. Like other cell culture systems, intestinal organoids lack the full spectrum of biochemical and biophysical cues observed *in vivo.* As consequence, some studies have transplanted intestinal organoids under the kidney capsule of immunodeficient [9, 11] or humanized mice [95] to overcome this limitation. While immune cells infiltrate these engrafted organoids and form lymphoid structures [95], the accessibility and control over immune–epithelial interactions offered by *in vitro* immune-organoid co-cultures are lost upon transplantation. Continuous efforts are being made to optimize the *in vitro* biochemical cues and physical properties of the gut can be modelled using modifiable synthetic gel matrices as an alternative to commonly used animal-derived ECM-rich gels [23, 96, 97]. Despite these limitations, intestinal organoids currently provide the most accurate *in vitro* system of the intestine and have allowed reductionist investigation of immune–epithelial interactions in the gut, demonstrating great potential as a tool for both fundamental and translational research.
